# Susceptibility of Avian Species to Brucella Infection: A Hypothesis-Driven Study

**DOI:** 10.3390/pathogens9020077

**Published:** 2020-01-24

**Authors:** Gamal Wareth, Ahmed Kheimar, Heinrich Neubauer, Falk Melzer

**Affiliations:** 1 National/OIE Reference Laboratory for Brucellosis, Friedrich-Loeffler-Institut, Institute of Bacterial Infections and Zoonoses, Naumburger Str. 96a, 07743 Jena, Germany; 2 Department of Pathology, Faculty of Veterinary Medicine, Benha University, Moshtohor, Toukh 13736, Egypt; 3 Department of Poultry Diseases, Faculty of Veterinary Medicine, Sohag University, Sohag 82524, Egypt

**Keywords:** avian, brucellosis, susceptibility, transmission, reservoir, serology

## Abstract

Brucellosis is a highly contagious bacterial disease affecting a wide range of animals, as well as humans. The existence of the clinically diagnosed brucellosis in avian species is controversially discussed. In the current study, we set to summarize the current knowledge on the presence of brucellae in avian species. Anti-*Brucella* antibodies were monitored in different avian species using classical diagnostic tools. Experimental infection of chicken embryos induced the disease and resulted in the development of specific lesions. Few empirical studies have been performed in adult poultry. However, the isolation of brucellae from naturally-infected chickens has not been possible yet.

## 1. Introduction

Brucellosis is a significant cause of zoonosis, with worldwide distribution caused by the genus *Brucella* (*B.*). To date, the genus *Brucella* contains 12 accepted nomo-species affecting terrestrial and aquatic mammals. *B. melitensis, B. abortus, B. suis, B. canis, B. ovis,* and *B. neotomae* have been primarily isolated from small ruminants, cattle, pigs, dogs, sheep, and desert woodrats, respectively [1]. Two species of marine origin have been described: *B. pinnipedialis,* affecting pinnipeds, and *B. ceti,* affecting cetaceans. *B. microti* have been isolated from the common vole (*Microtus arvalis*) [2,3], and *B. inopinata* have been isolated from a breast implant wound of a North American female patient [4]. Recently, *B. papionis* have been isolated from baboons (*Papio* spp.) [5] and *B. vulpis* from red foxes (*Vulpes vulpes*) [6]. Most of them are host preferential but zoonotic pathogens. *Brucella* is a facultative intracellular stealthy pathogen affecting a wide range of domesticated and wild animals, as well as humans. It can escape recognition of the innate immunity and evade intracellular destruction [7]. The virulence of *Brucella* spp. in a particular host species is mainly related to their intracellular replication. Their virulence depends on their survival and replication properties inside the host cells, as well as their ability to survive inside phagocytic and nonphagocytic cells [8]. *Brucella* displays a marked tissue tropism for the lymphoreticular and reproductive tract, causing significant clinical disorders and pathology. The intracellular lifestyle of *Brucella* limits exposure to innate and adaptive immune responses [9]. The pathogen is transmitted to humans by direct contact with diseased animals and excreta, e.g., during obstetrics or slaughter. It is thus an occupational risk in slaughterhouses and on dairy farms. The most common way of spillover hosts, however, is the ingestion of contaminated milk or milk products. Human-to-human transmission of brucellosis can occur via lactation, transplacental, and sexual routes, as well as by tissues, blood, and bone marrow transplantations, but it is rare [10]. 

In many developing countries, the disease is endemic and is a public health problem due to the high prevalence in livestock. Albeit the existence of highly restrictive control programs, some of these countries cannot implement these eradication programs effectively, resulting in the spreading and infection of nonspecific hosts [11]. Brucellae-infected livestock are host-restricted. For instance, *B. abortus, B. melitensis, B. suis,* and *B. ovis* preferentially infect cattle, small ruminants, pigs, and sheep, respectively [8]. It has been previously shown that *B. melitensis* can even be isolated from Nile catfish in Egypt [12] following the disposal of abort materials in the Nile and its canals. Furthermore, *B. abortus* was recovered from a dog and a cat after ingestion of infected placenta materials and ingestion of milk from *Brucella*-positive cows [13]. A *B. microti*-like organism was isolated from a domestic marsh frog (*Pelophylax ridibundus*) and the surrounding environment in a commercial farm in France [14,15]. A novel *Brucella* strain similar to a *B. inopinata*-like sp. was isolated from captive waxy tree frogs (*Phyllomedusa sauvagii*) and captive Colorado river toads (*Incisions alvarius*) [16,17]. These results show that *Brucella* can colonize in amphibians and persist in their environment. In addition, a novel ’atypical’ *Brucella* strain was isolated from a blue-spotted ribbon tail ray (*Taeniura lymma*) [18]. Natural *Brucella* infection in saltwater fish and amphibians extends the host range of this pathogenic bacterium. The ubiquitous distribution of brucellae in several reservoirs is the factor determining its global prevalence on all continents and in the majority of countries [19]. In the last decades, anti-*Brucella* antibodies have been found in different avian species, e.g., chickens, pigeons, and ducks, in some regions of Africa and Asia [20,21,22,23,24,25]. However, the pathogen has not been isolated yet from birds. Several studies have shown that wild and migratory birds are able to carry pathogens and serve as mechanical vectors or reservoirs for numerous infectious agents [26,27]. Recently, the examination of blood samples collected from 33 migratory birds along the Mediterranean revealed the presence of *Brucella* spp. in one sample obtained from the great reed warbler [28]. Thus, it is not clear whether poultry can be infected and induce a disease or may act as a susceptible host for brucellae, i.e., whether bacteria survive and replicate within birds and cause symptoms or whether they may only seroconvert. Consequently, no descriptions of specific pathological lesions are available. If poultry have to be considered as hosts, their role in transmission and dissemination has to be determined. In this report, we summarize previously published information on brucellae in avian wards and assist in solving the current debate.

## 2. Serological Evidence of Brucellosis in Birds

Until now, several bird species have been found to harbor anti-*Brucella* antibodies using the standard serological tests. The majority of these birds were kept in traditional husbandry systems with multiple animal species, e.g., small ruminants or cattle. This cohabitation system enhances potential transmissions from animals. A minimal number of studies [20,21,22,23,24,25] were performed in different countries to investigate whether poultry can harbor the *Brucella* organism (Table 1). Recently, Ali and colleges collected blood samples from 79 different avian species in Pakistan. The use of the Rose Bengal test (RBT) showed that two samples from a peafowl (*Pavo cristatus*) and an Indian blue rock pigeon (*Columba livia*) were positive [20]. A serological survey of traditionally managed 556 local chickens, 84 guinea fowl, 50 ducks, and 40 turkeys in Borno State, North-Eastern Nigeria using the RBT and the microtiter agglutination test (MAT) showed different degrees of seroprevalence. 1.8%, 7.1%, and 5% were positive in the RBT, and 2.3%, 9.5%, and 6% were positive in the MAT in chickens, guinea fowl, and ducks, respectively [24]. 2.8%, 2.3%, and 1.9% of blood samples collected from 355 pigeons, 510 chickens, and 255 Muscovy ducks in Nigeria were positive in the RBT, respectively [21]. Only one out of 150 blood samples collected from apparently healthy local chickens in Nigeria showed a positive reaction with the RBT [25]. 2.8%, 2.6%, and 3% out of 1000 blood samples collected from apparently healthy local chickens in North-Western Nigeria showed positive reactions with the RBT, slow agglutination test (SAT), and enzyme-linked immunosorbent assay (ELISA), respectively [22]. This poultry had been raised with ruminants, and the authors assumed that poultry might act as a mechanical vector for the transmission of the pathogen only [22]. In Botswana, Mushi and colleges have collected 220 serum samples from apparently healthy (backyard) indigenous chickens. The RBT and SAT revealed that two samples contained anti-*B. abortus* antibodies [23]. All of these studies were done without further confirmation by cultures, the gold standard for the diagnosis of brucellosis. It is highly likely that a significant crossreaction may be noted with other Gram-negative bacteria, e.g., *Salmonella* spp. or *Escherichia coli* O157 [29], which are known to be prevalent in poultry. Hence, the detection of anti-*Brucella* antibodies in birds is alarming and must be handled with care. Table 1 summarizes the results obtained in serological studies about brucellosis in birds that have been performed in different countries. 

## 3. Experimental Inoculation of Chicken Embryos with Brucellae

**Figure 1:**
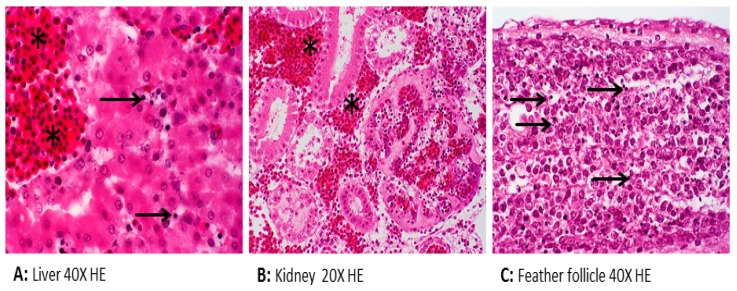
Histopathology of chicken embryos inoculated with *Brucella (B.) microti* showing apoptosis (**arrow**) and hemorrhage (**asterisk**).

**Figure 2:**
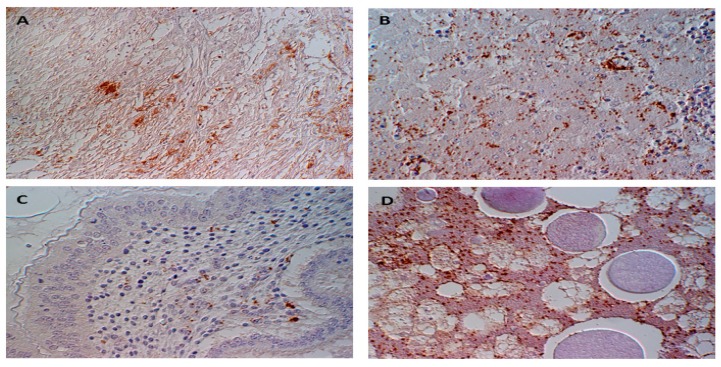
Immunohistochemical detection of *Brucella* sp. (stained brown) within and between (**A**) cardiomyocytes, (**B**) hepatocytes, (**C**) the lamina propria mucosae of proventriculus, and (**D**) cells of the yolk sac mesoderm.

To determine if brucellae can be propagated and replicated within chicken cells, experimental inoculation of chicken embryos was performed. One hundred chicken embryos (CE) were inoculated with milk from *B. abortus*-positive cows. At least 10% of inoculated embryos showed damage in liver cells and chorioallantoic membranes, i.e., necrosis and inflammation [30]. Detilleux and colleagues inoculated 125 CE with *B. abortus* via three different routes: yolk sacs, chorioallantoic membranes, and intravenously [31]. Electron microscopy analysis revealed the spread of bacteria to all tissues in all inoculated CEs. The intracellular localization of *Brucella* within the rough endoplasmic reticulum of mesenchymal, endodermal, and hepatic cells was evident. The liver, spleen, and heart were the most severely affected organs [31]. We previously demonstrated that *B. microti* replicated efficiently and induced pathological lesions in infected chicken embryos at day 11 of age [32]. Re-isolation of bacteria from allantoic fluid indicated the rapid multiplication of bacteria, and infection resulted in 100% mortality. *B. microti* provoked marked gross lesions, mainly hemorrhages, and necroses between days 2 and 4 post-inoculation. *Brucella*-specific lesions, such as prominent necroses and apoptosis in the liver, kidneys, lungs, spleen, spinal meninges, feather follicles, and chorioallantoic membrane, were the predominant histopathological lesions (Figure 1). *Brucella* antigen was demonstrated in nearly all of these organs by immunohistochemical examination (Figure 2). Even though the ability of *B. abortus* and *B. microti* to infect and to replicate within chicken embryos was proven very efficiently, the behavior of other *Brucella* spp. have not yet been assessed. Therefore, we assume that embryonated eggs are an outstanding substrate for the cultivation of brucellae. In contrast, no conclusions on the status of adult birds have been approved. The chicken embryo is known as one of the most useful animal tissues for the isolation of fastidious bacteria and viruses. However, using embryonated eggs as a standardized model in pathogenicity studies of brucellae instead of the laboratory animal mice needs more investigation.

## 4. Experimental Infection of Adult Birds with Brucellae

Studies have been performed on chicks and adult chickens to confirm that brucellae can survive within the adults and not only within embryos. It has been demonstrated that in one-day-old chicks, *B. abortus* infection provoked no prominent clinical signs, albeit the microorganisms recovered from two out of ten infected chicks [33]. Unlike *B. abortus*, the infection with *B. melitensis* resulted in the appearance of clinical symptoms 48–72 h post-infection, resulting in high mortalities. The microorganism was isolated from 6 out of 10 infected chicks. Layers experimentally infected with *B. melitensis* showed no apparent clinical symptoms, except for a slight decrease in egg production. Moreover, *Brucella* was recovered from the droppings, egg yolks and whites, and internal organs of some birds as well [33]. It is worth to mention that the microorganisms recovered from the infected chicks were identical to the inoculum strains.

In contrast, Kumar et al. failed to isolate *Brucella* organisms from lungs, liver, spleen, and fecal samples of experimental chickens infected with *B. abortus* [34]. It can be speculated that *Brucella* infections might be below the limit of detection in the tissues, despite detectable antibody titers. Oseguera Montiel assumed that scavenger birds that remove and eat infected materials could transport brucellae over considerable distances [35], either by moving it away or as a contaminated vector. Thus, animal houses must be disinfected, and placenta, aborted fetuses, and the dead animal carcasses must be hygienically disposed of to preclude spread.

In the last century, natural infections of fowl with *Brucella* and transmissions of diseases from aborting cattle to fowls were discussed several times [36,37,38,39,40]. Birds often show no clinical signs of illness, but when they do occur, symptoms frequently include enteritis and diarrhea [41]. Since no proof of morbidity has been demonstrated, further extensive studies are required to confirm the exact role of chickens and other avian species in the lifecycle of the *Brucella* and what the potential threat is to humans. Recently, fecal and blood samples have been collected from birds migrating along the Mediterranean and were investigated as carriers of zoonotic pathogens. The PCR assay revealed that *Brucella* spp. DNA was found in one (0.15%) blood sample [28].

## 5. In Vitro Infection of Chicken Macrophages with Brucellae

**Figure 3:**
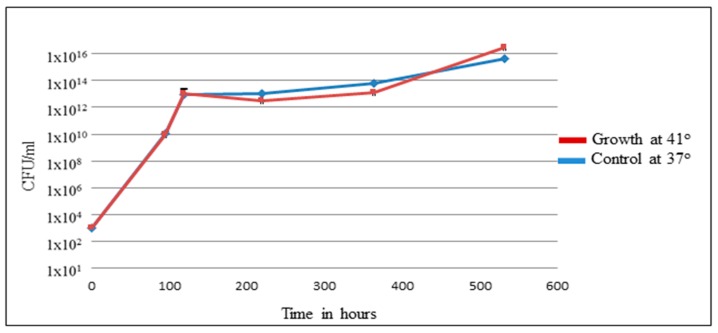
Survivability and growth curve of *B. microti* at 41° and 37°.

Brucellae are intracellular infectious agents with cell tropism mainly limited to the mononuclear phagocytic cells, preferentially macrophages [42]. *Brucella* does not produce classical virulence factors, such as exotoxin, plasmids, fimbria, and cytolysins. However, it can survive and multiply efficiently within both phagocytic and nonphagocytic cells [43]. *Brucella* infects hosts by adhering to and penetrating the mucosal epithelium surfaces. It has an initial adaption period inside epithelial cells, followed by a replicative phase [44]. The pathogenesis of *Brucella* infections is influenced by host factors, *Brucella* spp., and the ability to invade and survive inside macrophages. Persistence of *Brucella* inside macrophages represents essential strategies to evade the host immune response, complicate diagnosis, and may affect innate and adaptive immune responses [45]. To understand avian host responses to brucellosis, the direct bactericidal effect of a high temperature (41 °C) on *Brucella* was tested within avian macrophage HD11 [46]. It has been shown that at 41 °C, chicken HD11 macrophages infected with *B. abortus* resulted in a significant reduction of the intracellular replication of *Brucella* when compared to incubation at 37 °C, confirming the bactericidal effect of high temperatures [46]. However, high temperatures do not affect the rate of bacterial uptake, and no significant difference in the expression of target genes was observed. To elucidate the ability of *Brucella* to survive at 41 °C, in vitro culturing of *B. microti* in broth at 37 °C and 41 °C was performed for 500 h. As shown in Figure 3, the growth curve and survivability of *B. microti* at both temperatures were similar. The experiment was done with *B. microti,* which was isolated from common voles (*Microtus arvalis*). The intrinsic resistance against elevated temperatures demonstrated may enable brucellae to survive in avian hosts. MacDiarmid (1983) described the ability of brucellae to survive in fowl for up to two months. This assumption follows our in vitro experiment [41]. Hence, no proof for the avian clinical disease can be drawn from data of the literature or the laboratory. 

## 6. Conclusions 

Brucellae are Gram-negative facultative intracellular pathogens. The pathogens have been isolated from multiple aquatic and terrestrial mammalian hosts. The natural infection of nonspecific hosts extends the host range of brucellae and plays a significant role in global distribution. Despite the anecdotal association of brucellosis outbreaks in livestock and contact with scavenging birds, *Brucella* isolates have never been gained from adult birds raised with infected mammal hosts. All previous attempts that have been performed to detect anti-*Brucella* antibodies in birds were done without confirmation by cultures and molecular assays. However, it is alarming and must be handled with care. Cytoarchitectural damages in chicken embryos induced after the injection of brucellae have demonstrated the proliferation and pathogenicity of *Brucella* in chicken embryos. However, the infection of adult birds in animal trials gave no proof of the clinical disease. DNA of *Brucella* spp. have been identified in migratory birds. This work brings up the speculation that brucellae may use birds as ’mechanical’ vectors, or indeed, birds may serve only as short-term vessels of replication that can contaminate the environment. Therefore, the role of birds in the epidemiology of brucellosis needs to be evaluated. Further extensive studies are required in order to investigate the ability of *Brucella* to replicate within adult chicken macrophages and the biological consequences of this replication in terms of pathogenesis. Nevertheless, at least, birds in close contact with infected herds should not be ignored as potential carriers of brucellosis.

## Figures and Tables

**Table 1:** Results of serological studies about brucellosis in birds.

Bird species	Country	Number	Method	Positive	Yr. of Report	References
Turkey, Peafowl, Guinea Fowl, Mallard Ducks, and Indian Blue Rock Pigeon	Pakistan	79	RBT	2.5%	Ali et al., 2018	[20]
Local Chickens	Nigeria	556	RBT ^a^/MAT ^b^	1.8%/2.3%	Adamu et al., 2014	[24]
Guinea Fowl	Nigeria	84	RBT/MAT	7.1%/9.5%	Adamu et al., 2014	[24]
Ducks	Nigeria	50	RBT/MAT	5%/6%	Adamu et al., 2014	[24]
Turkey	Nigeria	40	RBT/MAT	-	Adamu et al., 2014	[24]
Pigeons	Nigeria	355	RBT	2.8%	Alaga et al., 2012	[21]
Chickens	Nigeria	510	RBT	2.3%	Alaga et al., 2012	[21]
Ducks	Nigeria	255	RBT	1.9%	Alaga et al., 2012	[21]
Chickens	Nigeria	150	RBT	0.75%	Gugong et al., 2012	[25]
Chickens	Nigeria	1000	RBT/SAT ^c^/ELISA ^d^	2.8%/2.6%/3.0%	Junaidu et al., 2006	[22]
Chickens	Botswana	220	RBT/SAT	0.9%	Mushi et al., 2008	[23]

^a^ Rose Bengal test, ^b^ microtiter agglutination test, ^c^ slow agglutination test, and ^d^ enzyme-linked immunosorbent assay.
